# Supplementary material for: A Humanized Yeast Phenomic Model of Deoxycytidine Kinase to Predict Genetic Buffering of Nucleoside Analog Cytotoxicity
Source: Genes (Basel). 2019 Sep 30;10(10):770. doi: 10.3390/genes10100770 (PMC6826991; doi:10.3390/genes10100770)
Supplement: Supplementary file 1 [file genes-10-00770-s001.zip › Additional_Files/Additional_File7_GOTermHeatmaps.zip!/Additional_File7_GOTermHeatmaps/histone-lysine_N-methyltransferase_activity.pdf]

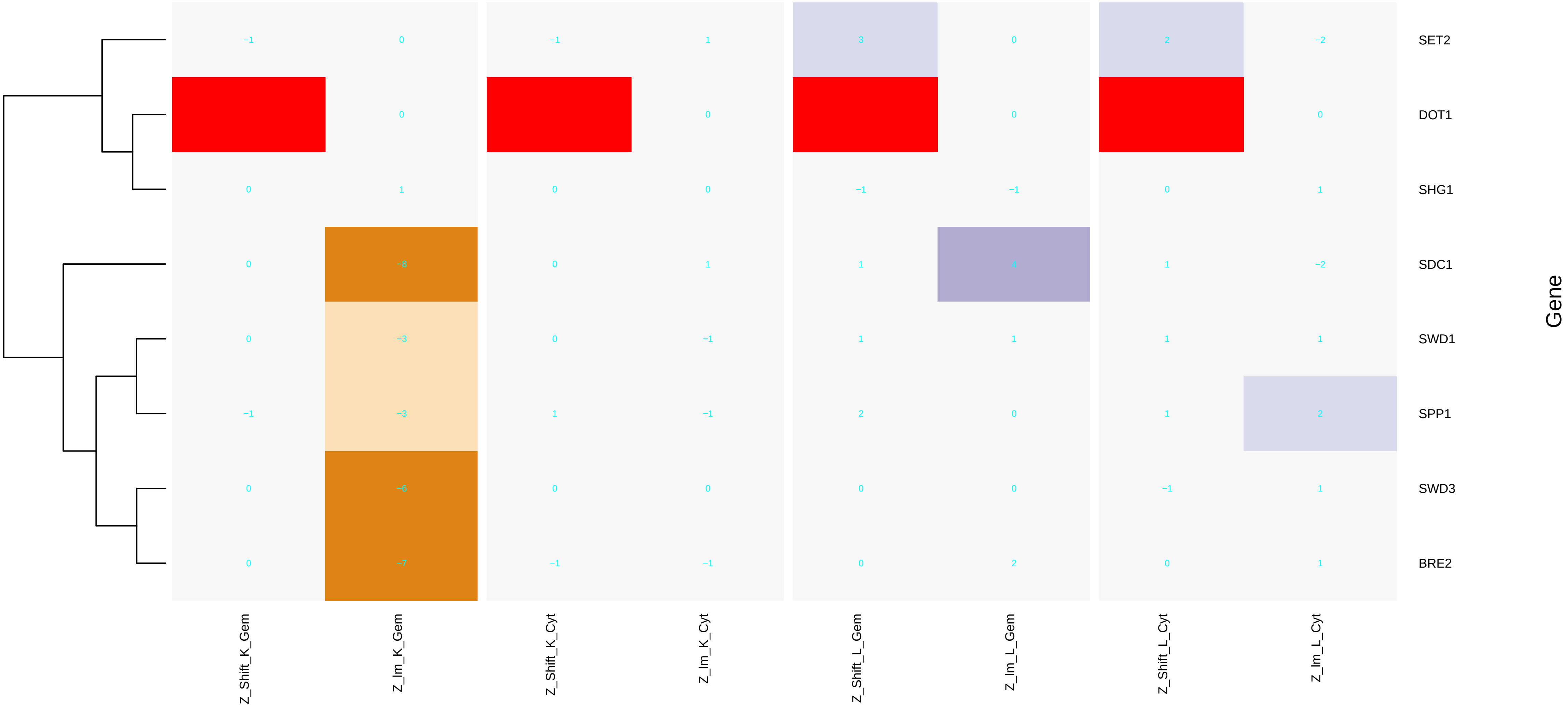

SET2 −1 0 −1 1 3 0 2 −2
DOT1 0 0 0 0
SHG1 0 1 0 0 −1 −1 0 1
SDC1 0 −8 0 1 1 4 1 −2
SWD1 0 −3 0 −1 1 1 1 1
SPP1 −1 −3 1 −1 2 0 1 2
SWD3 0 −6 0 0 0 0 −1 1
BRE2 0 −7 −1 −1 0 2 0 1
Z_Shift_K_Gem
Z_Im_K_Gem
Z_Shift_K_Cyt
Z_Im_K_Cyt
Z_Shift_L_Gem
Z_Im_L_Gem
Z_Shift_L_Cyt
Z_Im_L_Cyt
Gene
